# Pancreas lipiodol embolism induced acute necrotizing pancreatitis following transcatheter arterial chemoembolization for hepatocellular carcinoma: A case report and literature review

**DOI:** 10.1097/MD.0000000000018095

**Published:** 2019-11-27

**Authors:** Youwen Tan, Jianhui Sheng, Huiying Tan, Jianzhong Mao

**Affiliations:** Department of Hepatology, The Third Hospital of Zhenjiang Affiliated Jiangsu University, Zhenjiang, China.

**Keywords:** acute necrotizing pancreatitis, hepatocellular carcinoma, transcatheter arterial chemoembolization

## Abstract

**Rationale:**

Transcatheter arterial chemoembolization (TACE) is recognized as one of the most commonly used modalities for non-surgical treatment for advanced hepatocellular carcinoma (HCC). Ectopic lipiodol embolism is an extremely rare complication of TACE.

**Patient concerns:**

A 61-year-old man who had a 10-year history of cirrhosis caused by hepatitis B infection was diagnosed with ascites and HCC. Subsequently, the patient underwent TACE. However, he experienced persistent left upper abdominal pain, poor appetite, nausea, moderate fever and accompanied by elevation of serum and urine amylase on the 2nd and 3nd day after treatment.

**Diagnoses:**

The patient was diagnosed as having acute hemorrhagic necrotizing pancreatitis based on biochemical and inflammatory markers and CT findings. We deduced that the acute necrotizing pancreatitis was caused by a small branch of the left hepatic artery feeding the pancreas tail and embolizing the drug and lipiodol shunting to the tail of the pancreas.

**Interventions:**

The patient was treated for 5 days according to the comprehensive treatment of acute necrotizing pancreatitis, by the inhibition of the secretion of pancreatic juice, relieving pain, and total parenteral nutrition and forbidding diet. The symptoms of the patient were observed to improve, and SAMS and urinary amylase (UAMS) level decreased to 143 IU/L and 254 IU/L, respectively and oral diet was permitted.

**Outcome:**

After a period of 2 weeks, the contrast abdominal CT showed slightly decreased fluid collection of the peri-pancreatic space. Moreover, it also showed flocculous and linear high-density shadow in the pancreatic tail, suggesting lipiodol deposition in the pancreatic tail. Subsequently, the symptoms were observed to abate, and the patient left the hospital. On the 21st day after TACE, the patient had a follow up in our outpatient department; the biochemical characteristics and inflammatory markers were observed to be normal

**Conclusion:**

AP is still a rare complication after TACE. Etiology is still attributed to the occurrence of shunting and embolization drug reflux. Strategies strengthening the catheter tip that is placed as close to the distal branches of the hepatic artery for the possible careful injection of embolic materials is still the key to avoid post-TACE AP.

## 1 Introduction

At present, transcatheter arterial chemoembolization (TACE) is recognized as one of the most commonly used modalities for non-surgical treatment for advanced hepatocellular carcinoma (HCC).^[1]^ Postembolus syndrome is the most common adverse reaction of TACE treatment, mainly manifesting as fever, pain, nausea, and vomiting. The cause of fever and pain is local tissue ischemia and necrosis after hepatic artery embolization, and nausea and vomiting are mainly attributed to drugs used for chemotherapy.^[2]^

Ectopic lipiodol embolism is an extremely rare complication of TACE. In the TACE-induced ectopic embolization, cerebral embolism and pulmonary embolism have been reported.^[3–5]^ Although the incidence of acute pancreatitis (AP) after TACE has been reported to be ranging from 0.4% to 15.2%,^[6,7]^ acute necrotizing pancreatitis is still considered to be rare. However, the diagnosis of these cases of AP in the absence of evidence of pancreatic embolization or lipiodol deposition after TACE primarily comes from transient serum and urinary amylase elevations. In this study, we report a case of acute necrotizing pancreatitis by TACE which shows a clear evidence of pancreatic embolization and lipiodol deposition.

## 2 Ethics statement

Ethics Statement is not applicable for case report according to the Medical Ethics Committee of the Third Hospital of Zhenjiang Affiliated Jiangsu University, but Informed consent was obtained from the patient for publication of this case report and accompanying images. The study was conducted in accordance with the *Declaration of Helsinki*.

## 3 Case reports

A 61-year-old man who had a 10-year history of cirrhosis caused by hepatitis B infection was treated with entecavir for 5 years. He was brought to our outpatient department by his wife, who noted that he was complaining of upper abdominal discomfort for one month. Subsequently, he was diagnosed with ascites and a left liver mass by B-ultrasonography and was admitted to our inpatient department. The concentration of blood α-fetoprotein (AFP) was 46.6 μg/L, while the stage of hepatic cirrhosis was Child-Pugh B. His liver biochemistry tests displayed an elevated alkaline phosphatase level of 61 U/L, (ALP, reference range, 50–120 U/L), glutamine transpeptidase of 65 U/L (GGT, 10–40 U/L), alanine aminotransferase of 66 U/L (ALT, 10–40 U/L), and total bilirubin of 24.6 μmol/L (TBIL, 5–21 μmol/L), Serum albumin of 25.2 g/L (35–53 g/L), white blood cell (WBC) count of 3.7 × 10^9^/L, hemoglobin of 8.6 g/dL, hematocrit level of 27.9%, and platelet count of 46,000 per microliter of blood. A contrast abdominal computed tomography (CT) scan showed a 37 mm × 25 mm tumor in the left lobe of the liver with arterial phase hyper-enhancement and venous phase washout and the background of the liver appeared cirrhotic. Therefore, the patient underwent TACE treatment.

The TACE was performed via the left hepatic artery (LHA) using 5 ml mixed emulsification of epirubicin 10 mg and iodized oil 10 ml. Subsequently, lipiodol was deposited in the lesion and there was no significant intravascular reflux. Finally, embolization was performed with an appropriate amount of gelatin sponge pulp. On the 2nd day after treatment, the patient experienced persistent left upper abdominal pain, poor appetite, nausea, and moderate fever (38.1 °C). The patient's symptoms were treated conservatively with hydration, pain, and fever control, in accordance with post embolization syndrome. On the 3rd day, the patient also complained of left upper abdominal pain and fever (38.3 °C). His vital signs were stable and left upper abdominal examination revealed mild epigastric tenderness without palpable tender mass. The liver enzyme levels were similar to the levels before admission, but the WBC count 16.93 × 10^9^/L, C-reactive protein (CPR, < 8 mg/L) 50.34 mg/L, Procalcitonin 1.25 ng/L (PCT, 0.05–0.5 ng/L) serum amylase was 422 U/L (SAMS, 40 ∼ 110 U, Somogyi), urine amylase (UAMS) was 1244 U/L (80∼300 U/L, Somogyi) and serum calcium 1.85 mmol/L (Ca, 2.03–2.54 mmol/L.)

**Figure 1:**
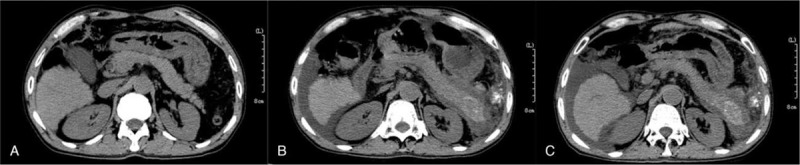
Abdominal CT image showing the pancreatic tail pre-and post-TACE. A: Normal pancreatic tail before TACE. B: Swelling and a low-density area in the tail of the pancreas, suggesting necrosis. Dense lipiodol accumulation in the dorsal pancreatic tail after the 3rd day of TACE. C: A flocculous and linear high-density shadow also appeared in the pancreatic tail, with a slightly low-density fluid effusion and a few spots of high density after 28th day of TACE.

A contrast abdominal CT revealed swelling and enlargement of the pancreatic tail, decreased density and non-uniformity. A flocculous and linear high-density shadow appeared in the pancreatic tail, with a slightly low-density fluid effusion and a few spots of high density (Fig. 1). As a result, necrotic pancreatitis was suggested. Therefore, the attending physician arrived at a diagnosis of acute hemorrhagic necrotizing pancreatitis (AHNP) based on the biochemical and inflammatory markers and CT findings.

The patient was treated for 5 days according to the comprehensive treatment of acute necrotizing pancreatitis, by the inhibition of the secretion of pancreatic juice, relieving pain, and total parenteral nutrition and forbidding diet. The symptoms of the patient were observed to improve, and SAMS and UAMS level decreased to 143 IU/L and 254 IU/L, respectively and oral diet was permitted.

After a period of two weeks, the contrast abdominal CT showed slightly decreased fluid collection of the peri-pancreatic space. Moreover, it also showed flocculous and linear high-density shadow in the pancreatic tail, suggesting lipiodol deposition in the pancreatic tail. Subsequently, the symptoms were observed to abate, and the patient left the hospital. On the 21st day after TACE, the patient had a follow up in our outpatient department; the biochemical characteristics and inflammatory markers were observed to be normal (Table 1).

**Table 1:**
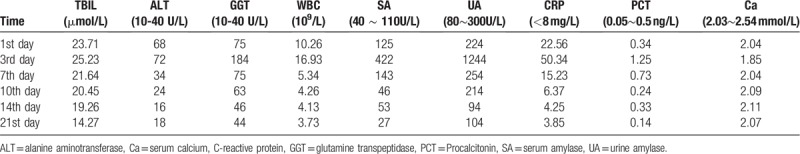
Biochemical characteristics and inflammatory markers in changes.

**Figure 2:**
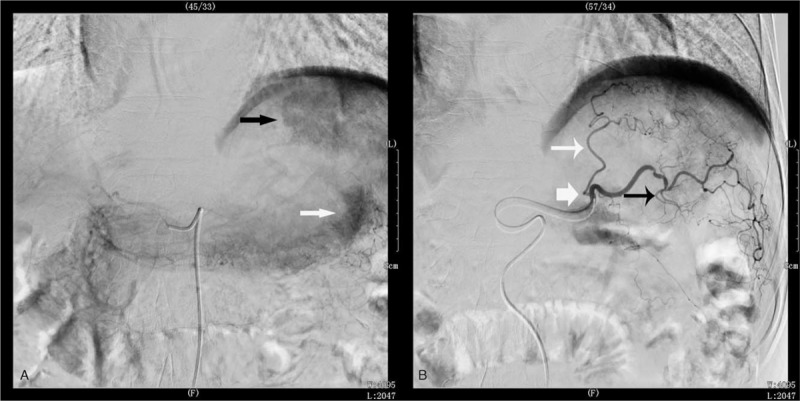
Liver imaging by digital subtraction angiography (DSA). A: A large lesion of HCC (black arrowhead) and pancreatic tail (white arrowhead); B: Micro catheter situated before the left hepatic artery bifurcation (white arrow). Lesion feeding artery from left hepatic artery (white arrowhead) and feeding pancreatic tail from left hepatic artery branch (black arrowhead).

Consequently, we deduced that the acute necrotizing pancreatitis was caused by a small branch of the left hepatic artery feeding the pancreas tail and embolizing the drug and lipiodol shunting to the tail of the pancreas (Fig. 2).

## 4 Discussion

TACE is currently widely used as a therapeutic method for HCC patients unsuitable for surgery or those with tumor recurrence after surgical resection.^[8]^ Moreover, TACE is also used as an adjuvant treatment before or after surgical resection.^[1]^ Hence, TACE has become the one of the important treatment modalities for advanced HCC worldwide.

The common complication of TACE, the post-embolization syndrome, is still an issue, including abdominal pain, vomiting, and fever. These symptoms can generally be resolved within a few days after the TACE.^[9]^ Extra-hepatic uptake of chemoembolization material in other organs is relatively common but usually does not cause any problems.^[10]^

Ectopic lipiodol embolism (ELE) is an extremely rare complication of TACE. In the TACE-induced ectopic embolization, iodized lung and cerebral embolism have been reported, although the incidence of acute pancreatitis (AP) after TACE has been reported to vary, ranging from 0.4% to 15.2%. We found 18 articles that reported 36 cases in a literature search^[6,7,11–27]^ from 1989 to 2017 are summarized in Table 2.

**Table 2:**
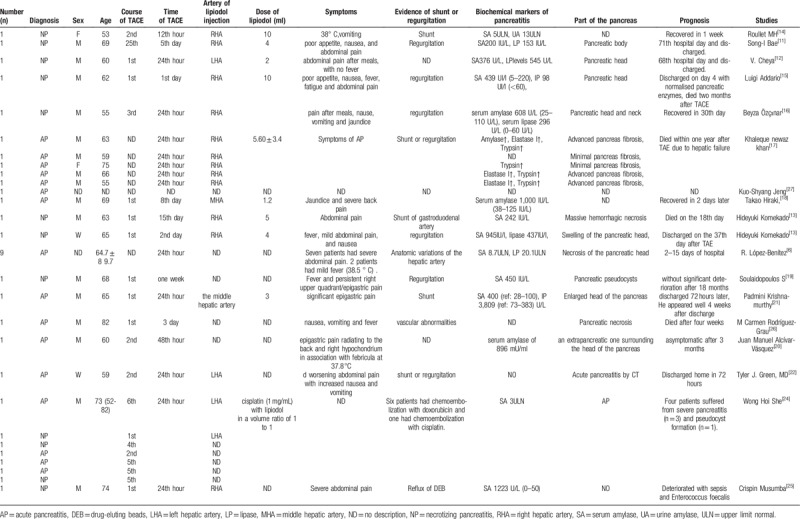
Summary of 36 cases of AP following TACE for hepatocellular carcinoma.

The diagnosis of AP was established mainly according to elevations of serum amylase and lipase levels, abdominal pain, and other symptoms. As reported in the literature, the diagnosis of acute pancreatitis relies on typical abdominal symptoms such as fever, abdominal pain, fatigue, vomiting, and elevation of serum amylase and lipase. Almost all of the reported cases were observed to be accompanied by elevations of serum amylase and lipase. Most of these abnormal findings occurred 24 hours after TACE. In our case report, the first detailed record of the process of necrotizing pancreatitis is caused by TACE embolization drugs except for elevations of serum amylase and lipase.

Re-elevation of pancreatic enzymes predicts worsening of the pancreatitis. Of the 23 reported cases of pancreatic injury, 7 were diagnosed as necrotizing pancreatitis and there was a lethal outcome when sepsis and multi-organ failure develop. Additionally, one among seven patients on the first post-TACE day complained of nausea, fever, and abdominal pain with an increasing value of alpha-amylase 439 UI/L (normal range 5–220), and lipase value of 98 UI/l (normal range <60). Moreover, there was no obvious pancreatic injury or ascites in the abdomen B-US. Moreover, 4 days later, the patient was discharged from the hospital due to normal serum amylase and lipase levels. However, 3 weeks later, the patient complained of severe abdominal pain and fever, and was re-admitted to the hospital. Consequently, abdominal US showed mild ascites in the patent portal vein. The patient gradually worsened owing to sepsis and multi-organ failure and eventually died two months after TACE.^[15]^

Chey et al^[12]^ reported a 60-year-old-male patient who exhibited AP by the elevation of serum amylase and lipase levels (376 U/L and 545 U/L, respectively) after TACE. Subsequently, an oral diet was commenced for 3 days until the lipasemia normalized. However, hyperlipasemia (1092 U/L) re-occurred on the 11th day, and CT showed NP in pancreatic head lesions. Therefore, an oral diet was recommenced until the lipasemia normalized. On the 36th day, there was a third peak of hyperlipasemia which was associated with a pseudocyst in the head of the pancreas. On the 68th day, the patient was discharged from the hospital as the CT scans showed a decrease in pancreatic lesions and normal lipase release.

Branch shunt or embolization regurgitation is the primary cause of pancreatitis. The Celiac artery branches shunt or embolization regurgitation plays an important role in pancreatic injury. Fortunately, such shunt and embolization regurgitation are usually not a significant problem as the dose deposited outside the liver is small. In the literature, the described symptomatic AP developed presumably because PVA particles regurgitated into the pancreaticoduodenal artery and occluded a high peripheral portion of the pancreatic vascular bed, leading to ischemia of the pancreas.

Serum amylase activity was also associated with various embolic materials; Wakahiko Kishimoto et al^[7]^ found serum amylase activity was increased slightly in the patients treated with chemotherapy alone or plus TAE with lipiodol and increased in all of the patients treated with chemotherapy plus TAE with gel-foam powder. She WH et al reported^[24]^ 7 (0.4%) patients suffering from AP from a single-center experience of over 1500 cases who underwent TACE 5434 times. A total of 6 patients had chemoembolization with doxorubicin, with 1 patient displaying chemoembolization with cisplatin. Hence, they considered doxorubicin eluting bead as a higher risk of acute pancreatitis (6/145 (4.1%) vs 1/1487 (0.1%), *P* < .0001). In our case report, the cause of necrosis and edema of the pancreatic tail is attributed to the embolic lipiodol shunting to branches suppling blood to the pancreas tail from the left hepatic artery and embolization.

## 5 Conclusion

Taken together, AP is still a rare complication after TACE. We should pay more attention to the occurrence of AP if patients sustained abdominal pain, fever, and elevated pancreatic enzymes occur within 24 hours of the operation. The progression of the disease to sepsis and multiple organ failure is the main cause of death, conventional pancreatic nutrition and antibiotic treatment is usually considered effective for post-TACE AP. However, etiology is still attributed to the occurrence of shunting and embolization drug reflux. Strategies strengthening the catheter tip that is placed as close to the distal branches of the hepatic artery for the possible careful injection of embolic materials is still the key to avoid post-TACE AP.

## Author contributions

**Data curation:** huiying tan.

**Funding acquisition:** Youwen Tan.

**Project administration:** Youwen Tan, jianzhong mao.

**Writing – original draft:** Youwen Tan, jianhui sheng.

**Writing – review & editing:** Youwen Tan.

Youwen Tan orcid: 0000-0002-5464-1407.
